# Exploring the Role of Reactive Oxygen Species in the Pathogenesis and Pathophysiology of Alzheimer’s and Parkinson’s Disease and the Efficacy of Antioxidant Treatment

**DOI:** 10.3390/antiox13091138

**Published:** 2024-09-20

**Authors:** Talin Gogna, Benjamin E. Housden, Annwyne Houldsworth

**Affiliations:** 1 Neuroscience, Clinical and Biomedical Sciences, University of Exeter Medical School, Exeter EX2 4TH, UK; tjeg201@exeter.ac.uk; 2 Living Systems Institute, Clinical and Biomedical Sciences, University of Exeter, Stocker Road, Exeter EX4 4QD, UK; b.housden@exeter.ac.uk; 3 Clinical and Biomedical Sciences, University of Exeter Medical School, Exeter EX2 4TH, UK

**Keywords:** Alzheimer’s disease, Parkinson’s disease, antioxidants, oxidative stress, reactive oxygen species, neurodegeneration, neuroinflammation

## Abstract

Alzheimer’s (AD) and Parkinson’s Disease (PD) are life-altering diseases that are characterised by progressive memory loss and motor dysfunction. The prevalence of AD and PD is predicted to continuously increase. Symptoms of AD and PD are primarily mediated by progressive neuron death and dysfunction in the hippocampus and substantia nigra. Central features that drive neurodegeneration are caspase activation, DNA fragmentation, lipid peroxidation, protein carbonylation, amyloid-β, and/or α-synuclein formation. Reactive oxygen species (ROS) increase these central features. Currently, there are limited therapeutic options targeting these mechanisms. Antioxidants reduce ROS levels by the induction of antioxidant proteins and direct neutralisation of ROS. This review aims to assess the effectiveness of antioxidants in reducing ROS and neurodegeneration. Antioxidants enhance major endogenous defences against ROS including superoxide dismutase, catalase, and glutathione. Direct neutralisation of ROS by antioxidants protects against ROS-induced cytotoxicity. The combination of Indirect and direct protective mechanisms prevents ROS-induced α-synuclein and/or amyloid-β formation. Antioxidants ameliorate ROS-mediated oxidative stress and subsequent deleterious downstream effects that promote apoptosis. As a result, downstream harmful events including neuron death, dysfunction, and protein aggregation are decreased. The protective effects of antioxidants in human models have yet to directly replicate the success seen in cell and animal models. However, the lack of diversity in antioxidants for clinical trials prevents a definitive answer if antioxidants are protective. Taken together, antioxidant treatment is a promising avenue in neurodegenerative disease therapy and subsequent clinical trials are needed to provide a definitive answer on the protective effects of antioxidants. No current treatment strategies have significant impact in treating advanced AD and PD, but new mimetics of endogenous mitochondrial antioxidant enzymes (Avasopasem Manganese, GC4419 AVA) may be a promising innovative option for decelerating neurodegenerative progress in the future at the mitochondrial level of OS.

## 1. Introduction

Neurodegenerative diseases continue to grow at alarming rates, they are characterised by progressive neuron death or dysfunction resulting in debilitating, life-altering disabilities and drastically lowering life expectancy. Alzheimer’s (AD) and Parkinson’s disease (PD) are amongst the most common neurodegenerative diseases ranking 1st and 2nd [1,2]. In the UK 944,000 and 153,000 people are estimated to live with dementia and PD respectively [1,2,3]. People living with dementia are expected to rise to 1.2 million by 2040 and 230,000 by 2045 for PD in the UK [1,2,4]. AD is the leading cause of dementia in the UK [2,5,6]. Current treatments revolve around alleviating symptoms, with the underlying cause of neurodegeneration left unaddressed [7,8]. Developing alternative treatment strategies that address neurodegenerative mechanisms of AD and PD that, may halt the progression or prevent the development of AD and PD is critical to achieve [7,8,9]. Successfully modulating neurodegenerative mechanisms in AD and PD could alleviate the progressive decline of patients afflicted with these diseases. This may decrease the ever-growing healthcare costs of caring for patients with AD and PD through halting or delaying progression of the diseases towards the advanced, severe stages of disease that require full-time care.

This literature review poses some important research questions, such as, can reactive oxygen species (ROS)-induced cytotoxicity be attenuated sufficiently to inhibit cytotoxic cell mechanisms; can ROS cytotoxic mechanism be inhibited enough to spare neurons and improve outcomes; and could antioxidant therapy be effective in slowing the progression of AD and PD? In answer to these research questions, the review outlines the role of ROS generation in the pathogenesis of AD and PD as well as evaluating the effectiveness of antioxidants in reducing ROS generation and neuron survival and function, as reported in research literature, suggesting the potential for treatments for AD and PD.

### 1.1. Reactive Oxygen Species and Oxidative Stress

ROS play an important role in the oxidative stress (OS) associated with neurodegenerative pathology. ROS are free radical atomic species that have unpaired electrons in uneven numbers. Examples of free radicals include oxygen radical, superoxide, hydroxyl, alkoxy radical, peroxyl, nitric oxide and nitrogen dioxide. Hydrogen peroxide, some nitrogen compounds and hypochlorous acid are examples of nonradical ROS. ROS are generated in the mitochondria and neutralised by antioxidant enzymes such as superoxide dismutase and catalase. Dysfunction of the antioxidant function in the mitochondria results in OS, which can cause the pathogenesis of neurodegenerative disorders [10,11].

OS as a factor in ND, in disorders, such as, AD, PD, Huntington’s Disease (HD), amyotrophic lateral sclerosis (ALS), multiple sclerosis (MS), and Fredrich’s Ataxia (FA) is well established where ROS is shown to alter the epigenetic landscape by gene methylation giving rise to aberrant epigenome changes.

Both endogenous and exogenous are a source of ROS, but the main very active endogenous ROS emanate from the mitochondria, where electrons are transported in the mitochondria in the reduction of O_2_ to H_2_O_2_ generating the superoxide anion radical, hydrogen peroxide and hydroxyl [11].

**Figure 1:**
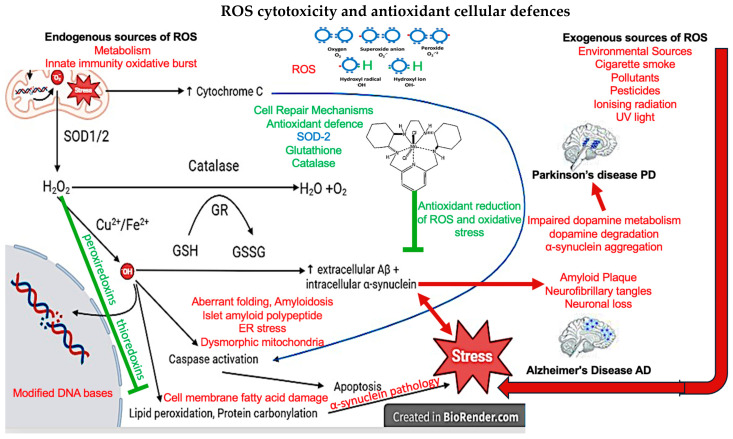
Diagram depicting the cytotoxic mechanism of ROS and cell defences against ROS. .O2 generated by aerobic respiration in which SOD1 converts .O_2_ in the mitochondrial matrix whilst SOD2 converts .O in the intermitochondrial space to H O . Then catalase breaks down H O into water and oxygen or H O oxidise glutathione into glutathione disulfide in the presence of 22 glutathione reductase. Cu^2+^/Fe^2+^ convert H_2_O_2_ into .OH, which oxidises DNA to cause double-strand or single-strand breaks. Thioredoxins and peroxiredoxins also maintain intracellular redox homeostasis reducing lipid peroxidation and the maintenance of cell membranes. Lipid peroxidation is important in the α-synuclein pathology associated with PD. .OH also activates caspases which break apart proteins that maintain the cell cytoskeleton, DNA repair, and ATP production as well as causing lipid peroxidation and protein carbonylation which causes cell stress. Lastly, .O_2_ damages mitochondrial DNA and causes the release of cytochrome C which activates caspases. Text in red indicates Abbreviations; .O : Superoxide anions, SOD: Superoxide dismutase, H_2_O_2_ : Hydrogen peroxide, .OH: Hydroxyl radical, GSH: Glutathione, GR: Glutathione reductase, GSSG: Glutathione disulfide, and Aβ: Amyloid-β.(9,10).

Superoxide anions, .O_2_ are formed when oxygen molecules are reduced from electrons provided by FADH2 and NADPH. Superoxide dismutase (SOD) converts .O_2_ into hydrogen peroxide (H O ) to mitigate free radical damage. Cu^2+^ and Fe^2+^ catalyse the breakdown of HO into hydroxyl radicals (.OH) and hydroxyl ions (OH-) through the Fenton and Haber-Weiss reactions [12] (Figure 1).

These highly reactive compounds that damage lipids, proteins, and nucleic acids through reduction-oxidation (REDOX) reactions, ref. [13] either donate (oxidation) or receive (reduction) electrons to regain stability [14]. Oxidation of lipids, proteins, and DNA by ROS, alters their unique structures that provide their function [13,14]. DNA damage is particularly harmful as DNA fragmentation induced by ROS is sufficient to initiate apoptosis [15] furthermore, DNA damage impairs the transcription and translation of vital proteins. Intracellular ROS are by-products of oxidative phosphorylation in mitochondria. FADH2 and NADPH are degraded by dihydrolipoyl dehydrogenases and NADPH oxidases respectively, to release electrons for the electron transport chain [14].

There are other sources of endogenous ROS including those generated by the host immune system, through inflammatory immune cell activation against infection and cancer [16]. ROS are generated by polymorphonuclear cells in an immune response and at sites of inflammation and this sometimes results in endothelial dysfunction and tissue injury. Also, mental stress, excessive exercise, aging and ischemia can be endogenous sources that generate ROS [17]. There are also some enzymic sources of ROS during phagocytosis as well as during an innate immune response as bactericidal ammunition during bacterial, virally infected cells, and fungal infections and can damage tissue indiscriminately [18].

Alternatively, exogenous generated sources of ROS emanate from alcohol, tobacco, smoke, pollution, heavy metals, transition metals, industrial solvents, pesticides, radiation and some drugs [17].

A number of chronic conditions can be triggered by exposures to environment ROS, and result in aberrant changes to the epigenome, remodelling of DNA methylation patterns occur. These changes of epigenetic reprogramming can occur and can be reversed by antioxidants [19,20].

Free radicals or free radical-generating compounds are eliminated by antioxidant proteins to protect cells against ROS-mediated oxidative stress (OS). Catalase, SODs, and glutathione comprise the main antioxidant defences against ROS, catalase catalyses the breakdown of H_2_O_2_ into water and oxygen, SODs convert .O into H_2_O_2_ and free radicals oxidise glutathione to gain stability. ROS elimination is vital for cell survival and impairments to antioxidant defences have deleterious consequences [21,22,23,24]. When the ratio of antioxidant defences to ROS production favours ROS generation OS ensues.

ROS promotes apoptosis through multiple mechanisms [12] and ROS fragment DNA, directly initiating apoptosis [14,15]. Increased Bcl-2-associated X protein (Bax)/B-cell lymphoma 2 (Bcl-2) ratio from OS initiates cytochrome C release from mitochondria. Bax forms pores on mitochondrial membranes whilst Bcl-2 forms heterodimers with Bax to prevent pore formation [15,25,26]. Caspases are proteases that break apart proteins involved in the cytoskeleton of cells, DNA repair, and ATP production [15]. Caspase activation therefore leads to the collapse of cell structure, rampant DNA damage, and reduced ATP production leading to the disassembly of a cell [15,22]. Upon sufficient cytochrome C release, caspase-9 is activated and subsequently activates caspases-3/5/7 [15]. Conversely, as previously described, ROS are involved in adaptations to stress, acutely raised levels of ROS can promote cell survivability, growth, proliferation, and enhanced antioxidant defences [12,13,15]. Lack of ROS can result in the arrest of the cell cycle and immune suppression [12,27]. Therefore, a delicate balance of ROS in cells is required for healthy physiological neural functioning (Figure 1).

### 1.2. Reactive Oxygen Species’ Role in Neurodegeneration

Neurodegeneration entails several mechanisms and ROS play a central role in mediating cell death and dysfunction in PD and AD [13,28,29,30].

There are large quantities of ROS generated in the brain due to it being a highly metabolically active organ, depending on oxidative phosphorylation as an energy source [31], and the delicate balance between antioxidant mechanisms and OS are significant factors in the pathogenesis of neurodegenerative disorders [32,33]. A high level of lipid is present in the CNS, and this consumes a high level of oxygen in a low antioxidant environment which makes the CNS particularly vulnerable to oxidative damage [33]. There is also a high metabolic demand for both neurons and microglia, which makes them more susceptible to OS. There is also a reduced rate of regeneration in these cells which makes the CNS more vulnerable to oxidative damage and the poor antioxidant potential leaves the CNS vulnerable to oxidative damage [34]. All of these sources of OS ultimately affect the survival and health of neurons and their survival, and many neurodegenerative disorders are associated with antioxidant mechanism dysregulation [33]. This acute imbalance between antioxidant function and the accumulation of ROS in the brain results in the OS that plays a principal role in the pathogenesis of neurodegeneration and the ROS generated gene mutations in mitochondrial DNA involved in cell metabolism [35].

Early studies on the neurotoxicity of free radicals have shown that knock-out (KO) of SOD2 resulted in immobility, severe motor symptoms (ataxia, paralysis, and tremor), and CNS degeneration [21,22]. As a result, SOD-depleted mice had a survival period no longer than 3 weeks. Building upon this Bhaskaran et al. recapitulated the neurotoxic effects of SOD2 KO in mature mice resulting in paralysis through OS [23]. Furthermore, other processes associated with OS were outlined such as neuroinflammation, increased inflammatory cytokines, and astrocyte and microglial activation [22]. Logan et al. outline that deficits in spatial learning and working memory can be induc3d by SOD1 KO [23]. Exposure to exogenous compounds such as rotenone, antimycin, and Fe^2+/3+^ is capable of inducing OS [36,37,38]. ROS-generating compounds induce or exacerbate pathological hallmarks of AD and PD such as α-synuclein and/or amyloid-β (Aβ) [37,38,39,40,41].

### 1.3. Pathogenesis of Neurodegenerative Diseases

Neurodegeneration is characterised by brain regions losing their structure or function through neuron death or dysfunction [9,42]. Neurodegenerative processes are involved in central nervous system (CNS) pathologies, from brain haemorrhages to multiple sclerosis [42]. Neurodegeneration in patients with AD or PD refers to, progressive cell death in conjunction with dysfunction of brain circuitry in the CNS [43]. In AD and PD significant cell death can be quantified by calculating volume loss in the brain. CNS atrophy in PD and AD manifests in separate brain regions, they each have unique pathophysiologies and pathogeneses with a portion of shared mechanisms [3,44,45,46].

### 1.4. Alzheimer’s Disease

The progression of AD is characterised into three stages: preclinical, mild cognitive impairment (MCI), and the dementia stage [47,48,49]. In the preclinical stages of AD, patients are asymptomatic but may have either low Aβ and/or increased hyperphosphorylated tau (pTau) in the CSF [50]. Activities of daily living (ADLs) remain unaffected in the preclinical stages of AD [48]. Mild dysfunction of memory, executive ability, and language function manifest in MCI stages of AD [49]. Progression to MCI does not guarantee progression to the dementia stages of AD, although, the probability of progression is significantly higher than the non-MCI population [51]. The dementia stage of AD consists of 3 stages: mild, moderate, and severe [48]. The ability to recall information and to get the day or week correct are the first abilities to be affected by AD [49]. In addition, this stage can be accompanied by impairments in problem-solving, judgement, executive functioning, lack of motivation, and disorganisation [52]. ADLs begin to be notably affected in the dementia stage [48,49]. Patients begin to have trouble assembling objects and drawing in the moderate stage of dementia. Significant problems with ADLs such as cooking, dressing, and eating also occur in the moderate stage [48,49]. Neuropsychiatric symptoms become more prevalent, notably psychosis, aggression, and disorientation, these symptoms contribute to the withdrawal of family support [48]. Patients in this stage cannot function adequately without close care from family/partners/carers [48]. The severe dementia stage is characterised by impairment to all cognitive functions with full impairments to attention, concentration, orientation in time and place, and immediate memory [48,49]. In the severe dementia stages of AD, a subset of patients may experience Parkinsonian symptoms, dyspraxia, olfactory dysfunction, dystonia, and akathisia [47]. Severe disinhibition in the final stages of AD can manifest as the inability to suppress primitive reflexes such as the grasp and sucking reflex [47,48]. Patients in the severe stage cannot communicate basic needs and require help with chewing and swallowing [48]. In AD life expectancy is reduced by a 3rd and the cause of death is either pneumonia followed by myocardial infarction or septicaemia [48].

In AD significant reductions in cortical volume first occur in the temporal lobe, particularly affecting memory-related structures including the entorhinal cortex and hippocampus [52,53,54,55]. Reduction in hippocampal volume and cerebral cortex atrophy with neuron loss in the temporal lobe gradually escalates in severity and migrates to additional regions as AD progresses [52,53,54]. Cortical degeneration in later stages of AD primarily affects cortical regions rather than subcortical regions [52,53,54]. All regions across the neocortex are subject to AD-mediated neurodegeneration or dysfunction except the precentral gyrus, which is in the parietal cortex [52,53,54]. The spinal cord and cerebellum are left spared [12,13,14]. Axonal degeneration may be an early event in AD pathology, connections between the hippocampus and central brain regions such as the cingulate gyrus are diminished in patients with MCI and AD [56]. Impaired communication between brain regions may further contribute to brain circuit dysfunction. The rate of neurodegeneration in AD is not exponential but rather follows a sigmoidal rate. Consisting of an early accelerating atrophy rate, plateauing, and deaccelerating atrophy rate [53,57]. Compound degeneration in AD results in severe dysfunction of brain circuitry with observable post- mortem changes in the structure of the brain [43,58].

There are some indications of familial inheritance of sporadic AD, where 40–60% are expressed in all AD patients. Of all the ApoE family (ApoE1-4), ApoE4 is the least functional antioxidant of the gene family and the least able to reduce OS [59].

Experimental demonstrations that explored the function of ApoE4 as an antioxidant in mice showed that an increased expression of antioxidant activity against OS was observed in animals with functional ApoE4 enzyme activity. Further, combinations of polymorphisms of SOD2 and ApoE4 are more prevalent in patients with amnesic MCI [60].

In addition, processes associated with AD pathology such as amyloid-β (Aβ) and tau pathologies are linked to an ApoE4 genotype, but also strongly associated with OS as a susceptibility gene for some ND and the pathogenesis of the AD phenotype [59,61].

### 1.5. Pathogenesis of Alzheimer’s Disease

AD pathology initiates, persists, and spreads by the interrelation between Aβ, neurofibrillary tangles (NFTs), ROS, and neuroinflammation [13,36,37,62,63]. AD may begin with a long period of OS in the brain before Aβ and pTau formation [64]. ROS-induced OS and Aβ formation and aggregation are directly interlinked as aberrant ROS generation is sufficient to significantly increase Aβ production [36,37,62]. β-secretase is the rate-limiting step in Aβ formation from amyloid precursor protein (APP) and aberrant ROS production increases the activity of β-secretase resulting in increased Aβ formation [43]. Alternatively, Aβ aggregates generate OS through the Aβ-mediated formation of ROS, fulfilling a positive feedback loop [36,37,62]. pTau is generated from the action of several kinases phosphorylating tau, pTau then aggregates into NFTs [63]. NFT formation may be modulated by the presence of Aβ [63]. Kinase phosphorylation of tau at multiple sites causes tau to disassociate from microtubule- associated tubulin [63]. Tau is integral for the stabilisation of microtubules and dissociation can result in microtubule collapse [63]. Microtubule collapse may be an additional source of tau deposition and subsequent phosphorylation. pTau then accumulates and aggregates into oligomers and lastly NFTs. Apoptosis through caspase activation and DNA fragmentation is initiated by the presence of NFTs. This is in part due to the oxidant properties of NFTs although, NFTs initiate DNA fragmentation and caspase activation through other undefined mechanisms [65,66]. Neuroinflammation is closely linked with Aβ, NFTs, and ROS as aberrant ROS increases inflammatory cytokine levels and glial cell activation [23] Microglia and astrocyte activation results in inflammatory cytokine release such as tumour necrosis factor α (TNFα), interleukin (IL) 1β and IL-6 [39]. High inflammatory cytokine levels cause neuroinflammation and cell death, and TNFα action on TNFα receptors can result in apoptosis by caspase activation [35].

Propagation of Aβ and NFT spread throughout the brain has been characterised thoroughly via Thal and Braak staging respectively [44,46]. Aβ spread can be generalised as a movement of Aβ from the neocortex to the diencephalon, spinal cord, and cerebellum [44,46]. NFTs have a unique propagation pattern to Aβ and are characterised under Braak staging [44]. Braak staging describes NFT formation beginning in the hippocampus and propagating out from this region [44]. In the later Braak stages, NFTs are found in the majority of the neocortex with low deposition in the precentral gyrus [44]. Subcortical regions, notably the amygdala, thalamus, SNpc, and the hypothalamus have NFT deposition in the later Braak stages [44].

### 1.6. Reactive Oxygen Species Production in Alzheimer’s Disease

MCI precedes AD pathology, patients afflicted with MCI have a high chance of progressing into AD [67,68,69]. This stage is characterised by the absence of significant Aβ and NFT deposition and the presence of cognitive decline. This may be induced by an imbalance of ROS and antioxidant defence systems. OS alone reduces spatial learning and memory in mice [24]. Patients with MCI display significant OS through Increased copper content, impairments to the glutathione system, and reductions in vital antioxidant vitamins [70]. Significant Aβ and NFT deposition occur post MCI stages of AD, both of which increase the levels of ROS production [36,37,62,65,66,69]. OS is observed throughout the preclinical stages as well as the dementia stages of AD. Significant sources of ROS in the preclinical stages of AD are from mitochondria or exposure to exogenous oxidants, for example, pesticides rotenone and antimycin and ionising radiation (UV light). Whilst in post-MCI stages aberrant ROS production is mediated by Aβ and NFTs) [37,62,65,66].

### 1.7. Parkinson’s Disease

PD is commonly associated with motor symptoms, resting tremors, muscle rigidity, slow or decreased movement, or postural instability [71]. Autonomic and olfactory dysfunctions known as non-motor symptoms of PD present before the onset of motor symptoms [45,71]. Non-motor symptoms can include, decreased sense of smell, profuse sweating, sleep disorders, and bowel and bladder dysfunction [45,71,72]. Quantifying the severity of PD in terms of symptoms was first developed by Hoehn and Yahr (H&Y) in 1967 [73,74]. H&Y staging revolved around rating the involvement of motor symptoms across the body, dependence on assistance to carry out ADLs, and ability to walk [73,74]. Contemporary use of H&Y staging now involves new parameters of rating such as impairment of balance [73]. The unified PD Disease Rating Scale (UPDRS) is a more recent rating scale for PD. The UPDRS is separated into 4 sections and provides a more holistic appreciation of all aspects of PD including non-motor/motor symptoms of daily living, motor complications, and motor examinations [74], The UPDRS separates PD patients into mild, moderate, and severe PD and this correlates with neurodegeneration of the SNpc [75].

### 1.8. Pathology of Parkinson’s Disease

Neuron loss in the substantia nigra (SN) is the primary site of neurodegeneration relating to motor symptoms in PD [71,75,76]. The prodromal phase of PD is associated with non-motor symptoms including sleep disorders and olfactory and autonomic dysfunction [45,72,77,78]. Early α-synuclein aggregation and neurodegeneration in the olfactory bulb, locus coeruleus, and dorsal motor nucleus of the vagus nerve may underlie early prodromal symptoms in PD [45,79,80]. Resting tremors, akinesia, shuffling gait, and postural instability succeed neurodegeneration with the gradual decrease of dopaminergic neurons in the SN [75,76,79]. Specifically, neurodegeneration of dopaminergic neurons in the substantia nigra pars compacta (SNpc) that, project to the posterior striatum or lateral putamen [81,82]. Late stages of PD are associated with severe motor symptoms and compounded degeneration of the SNpc [75]. Non-motor symptoms of PD arise from cerebral shrinkage in other brain regions, for example, cognitive decline in patients with PD may occur from neuron loss in the locus coeruleus [78,83]. During the pathogenesis of PD nonspecific cortical atrophy with sulcal widening in addition to the enlargement of lateral ventricles characterise this condition. Other notable sites of neurodegeneration in PD are the hippocampus, amygdala, thalamus, and caudate nucleus [77,78,79]. Degeneration of SNpc is associated with alterations in the fronto-nigro-striatal dopamine D1-GABAergic direct pathway [84].

PTEN-induced kinase 1 (PINK1) and Parkin (PARK2) gene mutations are implicated in a pathogenic role in association with PD in an autosomal recessive inherited manner. The function of PINK1 involves protecting cell apoptosis that is induced by OS and may also be associated with other antioxidant factors as discussed later. PARK2 gene mutations affect programmed cell death mechanisms [85,86]. PARK7 DJ-1 encodes an atypical peroxiredoxin-like peroxidase that regulates transcription and is a redox-dependent chaperone. Peroxiredoxins, like thioredoxin, are key antioxidants that neutralise the hydrogen peroxide ROS that cause the generation of lipid peroxidation products, involved with synucleinopathies in the amygdala. This lipid peroxidation can cause cell membrane fatty acid damage. Figure 1. This thioredoxin system comprises of thioredoxin, NADPH and thioredoxin reductase as a defense system against OS and regulating dithiol/disulfide balance [87]. Mutations in PARK7 DJ-1 are associated with early onset PD in an autosomal-recessive inheritance [88].

### 1.9. Pathogenesis of Parkinson’s Disease

Neuropathological changes in PD present before the appearance of symptoms [72,78,89]. In humans, post-mortem histopathological staining reveals the deposition of misfolded α-synuclein and aggregation into Lewy bodies. This aberrant protein conformation of α-synuclein, mutation of Leucine-rich repeat kinase 2 (LRRK2) [90] and further factors that cause the dysfunction and impairment of mitochondria are some of the pathological causes of PD [4,44]. The spread of α-synuclein across the course of PD has been outlined, and the pattern of Lewy body formation was first described through Braak staging by postmortem analysis of PD brains [9,66]. Braak staging showed that the pattern of α-synuclein deposition first appeared in the olfactory bulb and spinal cord nuclei [45]. α-Synuclein propagates up through the spinal cord, into the midbrain then the diencephalon, and lastly migrates into the neocortex. Beach et al. built upon Braak’s work by linking propagation patterns of multiple Lewy-type synucleinopathies [89]. From this, it was shown that propagation of α-synuclein irrespective of disease largely followed Braak staging but with a divergence of pathways. From early stages, Lewy pathologies migrated into brain stem regions (medulla, pons, and substantia nigra) or limbic regions (amygdala, trans entorhinal cortex, and cingulate gyrus [46]. After these diversions Lewy pathology propagation converges in a way like Braak staging [46].

### 1.10. Reactive Oxygen Species Production in Parkinson’s Disease

A key neuropathological change in PD is the deposition of iron throughout the brain [4,36,91,92,93]. Increased iron concentrations due to dysfunction of iron homeostasis may play a key role in neurodegeneration and α-synuclein formation in PD [36,40,41]. Excessive generation of .OH through Fenton’s reaction causes extensive cellular stress and neuron death [36,39]. PD brains are associated with increased iron content in key regions relating to motor function including the SNpc, red nucleus, globus pallidus internus, and putamen [92]. Excessive iron accumulation occurs throughout the frontal, parietal, and insular cortices [93]. High concentrations of REDOX available iron (Fe^2+/3+^) are cytotoxic to cells. However, there is conflicting evidence that iron-induced aberrant ROS production is sufficient to induce α-synuclein formation [36,40]. What is certain is that dysregulation of iron homeostasis is a contributing factor to α-synuclein aggregation and neurodegeneration [36,40,41]. Specifically, greater iron accumulation is correlated with higher scores on the motor section of the UPDRS indicating more severe symptoms of rigidity, dyskinesia, postural instability, and resting tremor [91]. Iron-induced neurodegeneration in motor-related structures may result in significant dysfunction of motor symptoms, perceived as worse scores on the UPDRS. α-synuclein can induce aberrant ROS production resulting in DNA double-strand breaks, apoptosis, and reduced cell antioxidant defences [94]. In addition, α-synuclein can increase ROS levels [95].

### 1.11. Current Treatment of Alzheimer’s and Parkinson’s Disease

Clinical treatment of PD and AD revolves around symptomatic relief. For example, in AD current FDA- approved treatments are acetylcholine esterase (AChE) inhibitors, N-methyl-D-aspartate (NMDA) antagonists, orexin receptor antagonists, and Aβ monoclonal antibodies [7,8]. For PD, L-dopa, dopa decarboxylase inhibitors, catechol-O-methyltransferase (COMT) inhibitors, dopamine agonists, and monoamine oxidases (MAO) inhibitors are used to provide symptomatic relief [7]. In AD impairment to the cholinergic system, aberrant NMDA receptor activation, sleep disorders, and Aβ deposition are prevalent [7,8,47]. AChE inhibitors are prescribed to prevent the breakdown of acetylcholine and sustain physiological levels. NMDA antagonists prevent excessive activation of NMDA receptor activation by blocking glutamate binding. Sleep disorders are prevalent in a subset of patients with AD and orexin receptor antagonists to alleviate sleep disturbances outside of AD [7,8]. Aβ monoclonal antibodies have been recently approved by the FDA to target underlying pathological mechanisms of AD but results have been controversial with no measurable change in cognition [7]. In PD, depletion of striatal dopamine gives rise to motor symptoms, therefore dopamine supplementation is given in the form of L-DOPA. L-DOPA crosses the BBB and is converted into dopamine other treatments of PD increase striatal dopamine by increasing L-DOPA bioavailability or preventing dopamine metabolism. COMT converts L-DOPA to dopamine in the periphery since dopamine cannot cross the Blood-brain barrier (BBB) this lowers the bioavailability of L-DOPA therefore COMT inhibitors prevent L-DOPA conversion. MAO breaks down dopamine in the CNS therefore inhibitors prevent this breakdown and raise levels of dopamine in the CNS [7]. Another form of treatment for PD is dopamine agonists, which mimic the effects of dopamine and alleviate symptoms driven by dopamine depletion [7].

### 1.12. Antioxidant Treatments for Alzheimer’s and Parkinson’s Disease

Due to the presence of OS in AD and PD antioxidant treatments have been investigated. Early applications of antioxidant treatment looked at the effects of antioxidant vitamins [96,97,98,99]. However, the inconclusive results of antioxidant vitamin treatment have led to the investigation of other antioxidants [100,101,102,103]. Specifically, herbal extracts have garnered significant attention for their neuroprotective properties [104,105,106]. Attenuating ROS production has had success in attenuating inflammatory cytokines ROS generation and ROS-induced toxicity [37,66]. Antioxidants such as Polyphenols, carotenes, vitamins, estrogenic compounds, and iron chelators have been investigated for their effects on attenuating ROS levels and therapeutic effects in cellular models of AD and PD. Polyphenols have promising effects in aberrant ROS cell models as well as cell models of AD and PD.

Recent advancements have been made in producing mimetics of human antioxidant enzymes.

Antioxidant enzymes play a significant role in maintaining safe levels of OS in biological systems. One important antioxidant enzyme, as previously described, is SOD-2 and increased expression of this enzyme is protective against OS in mice. Direct damage to DNA, proteins and lipids. Several phenotypes are identified where the loss of SOD-2 expression is associated with pathology of disease [33,107]. Further to this, memory deficits were prevented in mice where a reduction of cortex and hippocampal superoxide radicals was reduced [24,33,107,108]. Supporting the enhancement of SOD-2 expression in the elderly may be suggested by the loss of manganese superoxide dismutase (SOD-2) expression that has been observed in humans in an age-related pattern reducing the physiological defence against ROS [109].

The administration of SOD-2 supplementation is not common in practice. There are complications for oral administration due to the protein being digested in the gut before it is able to act systemically as an antioxidant enzyme, so further understanding of the role of SOD-2 function, in reducing OS in humans needs to be better defined, especially in relation to neurodegenerative disorders like AD. New SOD-2 mimetics have been developed to reduce OS after radiotherapy in cancer patients, Avasopasem Manganese (GC4419 AVA), selectively reduces superoxide dismutase and peroxide in cancer patients [110]. thus, could exogenous supplementation of SOD-2 be administered in humans to reduce the OS that causes neurodegeneration and slow the progression of PD and AD [13].

## 2. Methods

**Figure 2:**
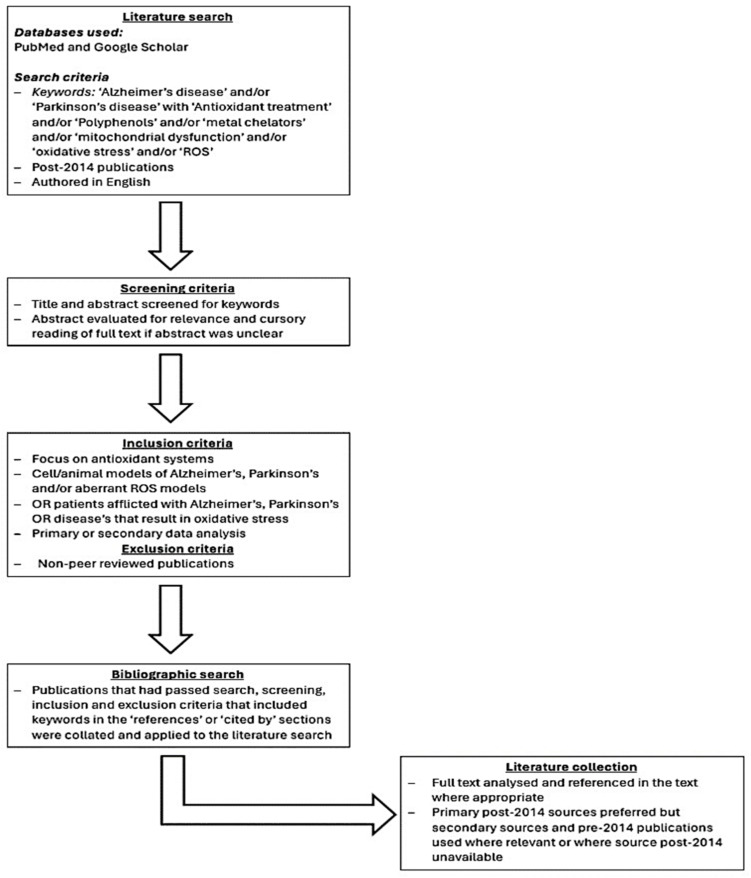
Flow chart depicting the search, screening, inclusion, and exclusion criteria in conjunction with the order followed. Papers collated from this method are referenced in the text were analysed, and relevant information was extracted and referenced where appropriate.

The literature primarily focuses on antioxidant vitamins, polyphenols, and carotenoids. Therefore, the literature search was primarily focused on gathering papers assessing their protective effects. Metal chelators and estrogenic compounds were also reviewed for their protective effects. Between October 2023 and March 2024, papers were put together using Google Scholar and PubMed following search, screening, and inclusion and exclusion criteria (Figure 2).

## 3. Discussion

### 3.1. Antioxidant Treatment in Cell Models of Alzheimer’s and Parkinson’s Disease

**Figure 3:**
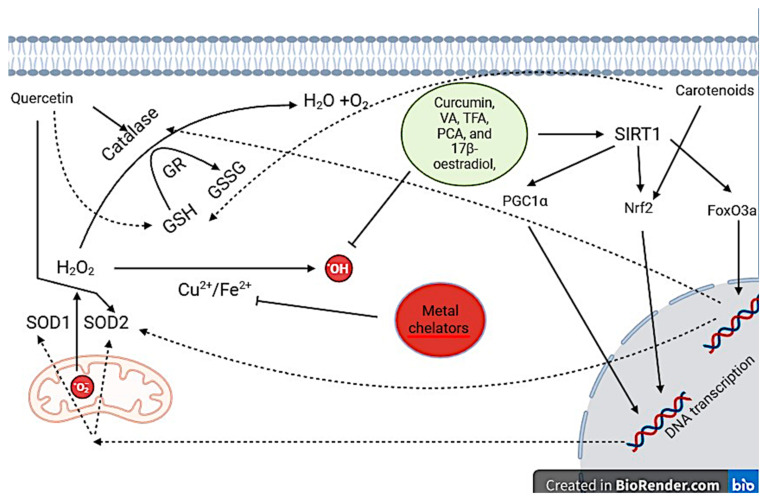
Cellular protective mechanisms of key antioxidant treatments. Curcumin, VA, TFA, PCA, and 17β-oestradiol have direct experimental evidence that they activate SIRT1. As a result, SIRT1 activates transcription factors by removal of acetyl groups: PGC1α, Nrf2, and FoxO3a. PGC1α and Nrf2 increase the expression of SOD1/2 whilst FoxO3a increases catalase expression. Other antioxidants quercetin and carotenoids increase antioxidant defenses they may act through SIRT1 activation although unclear, furthermore, they both increase glutathione expression. Metal chelators prevent the generation of .OH by binding to free Cu^2+^ and Fe^2+^ and preventing them from participating in REDOX reactions. Abbreviations: .O_2_: Superoxide anions, SOD: Superoxide dismutase, H_2_O_2_: Hydrogen peroxide, .OH: Hydroxyl radical, GSH: Glutathione, GR: Glutathione reductase, GSSG: Glutathione disulfide, SIRT1: Silent information regulator 1, PGC1α: Peroxisome proliferator-activated receptor gamma coactivator.

Attenuating ROS production has had success in decreasing ROS levels, ROS-induced toxicity, and inflammatory cytokines. Antioxidants such as Polyphenols, carotenes, vitamins, estrogenic compounds, and metal chelators have been investigated for their effects on attenuating ROS levels and therapeutic effects in cellular models of AD and PD. Polyphenols have been shown to have promising effects in aberrant ROS cell models as well as cell models of AD and PD (Table 1 and Figure 3).

The polyphenol quercetin is found in grapes, olive oil, apples, and blueberries. Primary and secondary antioxidant properties of quercetin protect SH-SY5Y and PC12 cells from ROS-induced cytotoxicity and neuroinflammation. When SH-SY5Y cells are exposed to Lipopolysaccharide (LPS), toll-like receptors (TLRs) are activated resulting in an inflammatory response. TLR activation results in an increased production of inflammatory cytokines which induce cytotoxic effects that significantly reduce cell viability [111,112]. Inflammatory cytokines Interferon-gamma (IFNγ) and interleukin-6 (IL-6) generate ROS by modulating the mitochondrial respiratory chain and NADPH oxidases [112]. As a result, increases in ROS induce OS which causes the initiation of apoptosis through direct and indirect pathways [15,112]. Treatment with quercetin ameliorates reductions in cell viability induced by LPS and OS [111,113,114]. This may occur through increased glutathione expression and its downstream effects including reducing inflammatory cytokines; IFNγ and IL-6, and direct ROS scavenging capability [111,113]. Glutathione may protect SH-SY5Y cells against inflammatory cytokine- mediated raised levels of ROS by scavenging free radicals generated by ROS. The primary antioxidant properties of quercetin may protect SH-SY5Y cells against ROS-mediated OS by quercetin directly scavenging free radicals released by ROS [113]. Direct and indirect scavenging of free radicals from quercetin prevents ROS from damaging proteins, lipids, and nucleic acids. This is supported by; quercetin treatment decreases markers of ROS-mediated oxidative damage such as DNA oxidation, lipid peroxidation, and protein carbonylation [111]. These results have been recapitulated in a PC12 cell model of PD where quercetin treatment protects against 6-hydroxydopamine (6-OHDA) mediated generation of free radicals and subsequent reductions in cell viability [114]. Notably, quercetin treatment reduces α-synuclein aggregation by decreasing aberrant ROS production [114].

Other polyphenols such as vallinic acid (VA), trans-ferulic acid (TFA), and protocatechuic aldehyde (PCA) mitigate the effects of H O -induced OS in SH-SY5Y cells primarily, through secondary antioxidant properties [25]. PCA, VA, and FA rescue H O induced decreases in cell viability and the number of ‘healthy’ SH-SY5Y cells [25]. These polyphenolic compounds protect SH-SY5Y cells against H_2_O_2_ cytotoxicity by reducing ROS levels from H_2_O_2_ [25]. A mechanism by which PCA, VA, and FA reduce ROS-induced OS is the activation of the enzyme silent information regulator 1 (SIRT1) [25,115]. Downstream removal of acetyl groups (deacetylation) from transcription factors by SIRT1 activates them so that gene transcription is initiated [115]. Targets of SIRT1 include forkhead box class O 3a (FoxO3a), peroxisome proliferator-activated receptor gamma coactivator 1-α (PGC1α), and nuclear factor erythroid 2-related factor 2 (Nrf2) [115]. FoxO3a activation results in increased SOD2 and catalase expression, PGC1α and Nrf2 also increase SOD expression and Nrf2 decreases the expression of inflammatory cytokines: TNFα and IL-1 (Nrf2) [115]. Catalase and SOD2 prevent ROS- mediated OS by neutralising .HO and free radicals [25]. Reduction of ROS reduces the number of cells going through apoptosis namely, by decreasing mitochondrial and nuclear damage. This is evidenced by the restoration of Bcl-2 levels and reducing free radical-mediated apoptosis pathways [25].

Sinapic acid, a phenolic acid present in citric fruits, has been shown to have protective effects in SH- SY5Y cells exposed to 6-OHDA [116]. Sinapic acid promotes restorations in cell viability when decreased by 6-OHDA [117]. Secondary antioxidant mechanisms activated by Sinapic acid play a significant role in protecting SH-SY5Y cells from 6-OHDA-mediated DNA fragmentation and apoptosis signalling [117]. Sinapic acid may modulate SIRT1 activation as, secondary antioxidant defences are initiated by downstream targets of SIRT1 such as PGC-1α, Nrf1, and TFAM [115,117]. Consequently, SOD1, 2, and catalase were expressed in greater numbers compared to 6-OHDA-treated SH-SY5Y cells [93]. Therefore, aberrant ROS generation from 6-OHDA was attenuated. Aberrant ROS generation is known to cause DNA fragmentation, caspase activation, and activation of the mitochondrial intrinsic pathway [15]. When SH-SY5Y cells were treated with Sinapic acid and 6-OHDA there was reduced DNA fragmentation, caspase activation, and activation of the mitochondrial intrinsic pathway [117].

Curcumin has been implicated as a protective agent in PC12 and SH-SY5Y cell models of PD [100,101,116]. Curcumin, which is a polyphenol found in turmeric, protects PC12 and SH-SY5Y cells against 6-OHDA toxicity [100,101]. Curcumin derivatives restore cell viability levels in part by attenuating ROS levels [100,101]. Curcumin may be another SIRT1 activator as it protects SH-SY5Y cells against aberrant ROS generation by increasing glutathione expression through the signalling of Nrf2 [101]. Successively, curcumin derivatives reduce α-synuclein aggregation in SH-SY5Y cells that overexpress α-synuclein by reducing ROS generation [40,41,101]. Interestingly, curcumin treatment had no effect on reducing Aβ levels when overexpressed in SH-SY5Y cells [101]. This may be from the type of model of AD used, preformed Aβ was directly exposed to SH-SY5Y cells, and Aβ oligomers were measured [101]. ROS may promote the generation of Aβ rather than oligomerisation therefore antioxidant treatment would have no effect on Aβ oligomerisation as shown here. The protective effects of curcumin have been recapitulated in PC12 cells treated with 6-OHDA, however, another protective mechanism is outlined [100]. Autophagy is a highly conserved process that involves the degradation of cytoplasmic components or organelles [118]. In aging, physiological ROS generation damages the DNA, proteins, and lipids of mitochondria so that they produce less ATP and more ROS, autophagy degrades damaged mitochondria to reduce OS [118]. However, α-synuclein aggregations can inhibit autophagy and in turn, result in impaired autophagy and excessive ROS production. Curcumin is shown to promote autophagy as shown through the marker of autophagy phosphatidylethanolamine conjugate (LC3-II) [102]. Lastly, curcumin in turmeric extract is shown to directly downregulate AD risk genes such as PSEN1, PSEN2, and APP in conjunction with promoting activation of SIRT1 [94]. Taken together, curcumin may protect cells against aberrant ROS production and α-synuclein aggregation through promotion of adaptive response to stress, initiation of autophagy, and downregulation of AD risk genes [100,101,116].

Iron and copper accumulation is involved in the pathogenesis of PD and AD, progressive increases of iron and copper in the brain result in cell cytotoxicity [70,92,93]. Fe^2+^ and Cu^2+^ generate .OH from H_2_O_2_ therefore, metal homeostasis is vital for neuron survival. metal chelators have been investigated for their possible neuroprotective properties in AD and PD and, are protective in SH-SY5Y cell models of PD [119]. Metal chelates such as 1-hydroxypyazin-2-ones (HPO) neutralise Fe^2+^ and by extension Cu^2+^ by binding to them and preventing them from generating free radicals from Fenton’s/Haber- Weiss’s reaction [120]. Several isomers of HPO protect SH-SY5Y cells from 6-OHDA neurotoxicity and significantly increase deficits in cell viability [120]. HPO treatments penetrated cell membranes and chelated REDOX available metals to produce protective effects [98]. Therefore, the use of metal chelates in the treatment of PD and AD may prevent REDOX available metals from generating free radicals and in turn neurodegeneration.

Oestrogenic compounds are tumour-suppressive through antioxidant mechanisms, this has generated interest in investigating their effects on neurodegenerative diseases such as AD and PD [121]. Treatment of 17β-oestradiol protects SH-SY5Y cells against high concentrations of D-galactose which generates ROS [121]. The protective effects of 17β-oestradiol are dependent on oestrogen receptor activation and SIRT1 activation [120]. SIRT1 activation drives enhanced antioxidant defences through the deacetylation of Nrf2, PGC1α, and FoxO3a [115,121]. Overall, 17β-oestradiol protects SH- SY5Y cells from aberrant ROS production by D-galactose by increased expression of antioxidant enzymes. In addition, depletion of oestrogen from menopause, ovariectomy, and hypothalamic dysfunction may confer increased susceptibility to OS and neurodegenerative diseases.

Vitamins play an important role in maintaining critical functions in the body such as immune function, bone integrity, and wound healing. They have been shown to have antioxidant properties. In this regard, they have been investigated for their antioxidant properties against ROS generation in AD and PD cell models. Vitamin B12 has been shown to have protective effects against ROS generation in SH-SY5Y cells [119]. Vitamin B12 can reduce aberrant ROS generation initiated by H O and increases cell viability. The protective effects of vitamin B12 are dependent on 22 polypyrimidine tract binding protein 1 which regulates apoptosis [119]. On the other hand, vitamin A has been shown to have deleterious effects in cell models of AD and PD. Aβ, α-synuclein and pTau aggregation is exaggerated by vitamin A in cells overexpressing Aβ, α-synuclein and pTau [99]. Although vitamin A the pro-oxidant effects and reductions of cell viability through ROS generation contribute to exaggerated Aβ, α-synuclein, and pTau aggregation, vitamin A exacerbates protein aggregation through aberrant ROS-independent pathways [99]. Currently, the way vitamin A increases Aβ, α-synuclein, and pTau aggregation is currently unknown [99].

Taken together, SIRT1 activation is a shared mechanism between antioxidants, particularly polyphenols that drives enhanced antioxidant defences protecting cells from Aβ and α-synuclein generation and subsequent aggregation. However, other mechanisms against ROS involve direct free radical scavenging and induction of autophagy. The combination of these mechanisms significantly decreases ROS formation and attack on cell structures, preventing cell death and dysfunction induced by ROS, Aβ, and α-synuclein. From this, the protective effects of these compounds depend on the presence of the compounds in the cell cytoplasm, and this asks the question of these compound’s ability to penetrate cell membranes and the BBB. Therefore, the protective effects of these compounds must be recapitulated in animal models to assess their validity.

### 3.2. Antioxidant Treatment in Animal Models of Alzheimer’s and Parkinson’s Disease

#### 3.2.1. Focus on Iron Chelating Mechanisms

Following preliminary success in cell models the protective effects of metal chelators were assessed in mice who were injected with iron dextran and 1-methyl-4-phenyl-1,2,3,6-tetrahydropyridine (MPTP) so that they were overloaded with iron [26,122]. Peripheral iron dextran injections increase free iron in the brain and reduce the activity of SOD and catalase. This increases the susceptibility of cells to ROS generation, resulting in the degeneration of neurons in the brain [26,122]. The chelating effects of hesperidin, coumarin, deferoxamine, and lactoferrin were assessed in mice with iron overload [26,122]. Hesperidin, coumarin, and deferoxamine reduce free iron levels in the periphery and brain [122]. As a result, deficits in SOD and catalase activity are recovered in the brain and periphery) [122]. Treatment of MPTP significantly increases free iron levels in the periphery and brain [48]. Treatment of lactoferrin can reduce iron levels in the periphery and, ameliorate deficits of SOD1 and 2 levels from MPTP treatment. As a result, lactoferrin treatment prevents the activation of the apoptotic mechanism, this includes raising Bcl-2 levels and reducing the amount of active caspase 3 [26]. Concerning PD lactoferrin reduces the amount of iron in the SN preventing iron-mediated neurodegeneration [26]. lactoferrin significantly increases tyrosine hydroxylase levels in the brain indicating the sparing of dopaminergic neurons in the SNpc [25]. Dopaminergic neuron protection through iron chelation was demonstrated by: iron overloaded mice treated with lactoferrin having restored ability to pole-climbing is significantly impaired without lactoferrin [26]. Copper and iron accumulation occurs in AD and PD, currently, no approved treatment addresses significant metal accumulation in the CNS [70,92,93]. From these results hesperidin, coumarin, deferoxamine, and lactoferrin are available in the CNS by crossing the BBB and mediating metal-induced cytotoxicity. Therefore, these compounds may provide avenues for addressing neurodegenerative mechanisms in AD and PD.

#### 3.2.2. Focus on Quercetin in Therapy

Following quercetin’s success in cell models its antioxidant properties were recapitulated in aberrant ROS and PD mice models [80,81,90]. Quercetin protects neurons from ROS-mediated neurodegeneration as quercetin prevents degeneration of dopaminergic neurons [114]. Quercetin significantly reduces astrocyte and microglial activation through decreasing IL-6, IL-1β, and TNFα levels [102,103]. Quercetin also significantly reduces ROS production and OS by increasing SOD levels [90]. In turn, the PD phenotype of reduced muscular strength, spatial learning, and spatial memory is improved following quercetin treatment [102,103,114]. The protective effects of quercetin were recapitulated in 6-OHDA-treated mice, these occurred through enhanced antioxidant defences, decreased neuroinflammation, and induction of autophagy [102,103,113]. Treatment of quercetin decreases α-synuclein aggregation by reducing ROS and consequently products of OS mediated, this likely was mediated by increased expression of SOD [114]. The decrease of neuron OS participated in the protection of dopaminergic neurons from ROS-cytotoxicity, as well as the induction of autophagy [114]. Autophagy is an important cellular mechanism that recycles damaged mitochondria, impairment of autophagy can result in an increased number of damaged mitochondria which produce less ATP and more ROS, so they contribute to cytotoxicity and α-synuclein aggregation [90].

Silencing of PTEN-induced kinase 1 (PINK1) resulted in reduced protective effects of quercetin for example, silencing of PINK1 resulted in significantly more α-synuclein aggregation (114). Lastly, quercetin protects mice against neuroinflammation [103]. LPS directly causes increased inflammatory cytokines through TLR recognition, inflammatory cytokines directly initiate ROS formation and apoptosis activation [15]. Quercetin treatment directly reduces apoptotic factors such as Bax and cytochrome C through reducing TNFα and IL-1β resulting in reduced cytokine signalling [103]. In addition, reduction of inflammatory cytokines and apoptosis attenuates aberrant activation of microglia and astrocytes which perpetuate an inflammatory response. Quercetin protected LPS-mediated neurodegeneration in the cortex and hippocampus resulting in improved spatial memory as seen through increased time spent in the target quadrant of the Morris water maze [105]. These results combined with quercetin effects in cells show that the primary protective mechanisms of quercetin encompass increasing glutathione and SOD expression and reducing inflammatory cytokines: IFNγ, TNFα, IL-6, and IL-1β [103,111,113,114]. Through this, these mechanisms reduce α-synuclein aggregation, apoptosis signalling, and OS [102,103,111,113,114]. Although Aβ levels were unmeasured it is appropriate to hypothesize that quercetin treatment could reduce Aβ levels.

#### 3.2.3. Focus on Carotenoids in Therapy

Carotenoids are another subset of non-enzymatic enzymes found in spinach, kale, and tomatoes and contain a long hydrocarbon-carotene chain with a ring of 6 carbons at either end. Carotenoids such as lutein and β-carotene protect mice from neurodegenerative mechanisms [123,124]. Lutein prevents dopamine depletion in the striatum by protecting dopaminergic neurons from MPTP- induced ROS damage [125]. Lutein restored deficits in glutathione levels incurred by MPTP thereby increasing protection from ROS [123]. Interestingly, SOD and catalase levels were not recovered and were significantly decreased when compared to the group of mice solely exposed to MPTP [102]. Glutathione levels were significantly raised in MPTP-treated mice, indicating the main protective effects were from glutathione and the primary antioxidant properties of lutein [123]. Therefore, reduced ROS generation reduces caspase activation and damage to mitochondria, resulting in reduced Bax and increased Bcl-2 [123]. Reduced degeneration of dopaminergic neurons from lutein treatment reduces the PD phenotype induced by MPTP. For example, treatment of MPTP induces characteristic PD symptoms such as rigidity, decreased locomotor activity, and impairments to motor balance and coordination of which treatment of lutein significantly improves these symptoms [123]. β-carotene protects mice from Aβ and ROS neurotoxicity [124]. Β-carotene improves recycling of the antioxidant protein glutathione possibly through increasing glutathione levels or decreasing ROS generation [124]. As a result, mice overexpressing Aβ have significantly reduced levels of Aβ rather than mice left untreated [124]. Following β-carotene treatment mice’s memory was recovered however, β-carotene showed both antioxidant and AChE inhibition so improvement may be due to the modulation of the cholinergic system which is already known to be impacted by AD and when modulated restores memory function [7,124].

Vitamin C possesses antioxidant properties and is a vital nutrient in diets [99]. In mice exposed to MPTP vitamin C protects mice against MPTP-mediated damage [101]. Specifically, MPTP treatment decreases dopaminergic neurons in the striatum and SNpc whilst, vitamin C prevents dopaminergic cell death to a certain extent [99]. The protective properties of vitamin C lie in its ability to reduce inflammatory cytokine levels such as IL-6 and TNF-α as well as increasing anti-inflammatory markers such as IL-10, Transforming growth factor beta, and IL-4. As a result, activation of microglia and astrocytes in the striatum and SNpc are significantly reduced [99]. Taken together reducing neuroinflammation induced by MPTP improves Parkinsonian mice phenotypes such as disturbed gait and decreases in locomotor activity [99].

#### 3.2.4. Focus on Flavonoids in Therapy

Flavonoids are a subset of polyphenols that have antioxidant properties, the flavonoid naringenin has been investigated for its antioxidant capabilities [125]. Iron overload in mice results in increased ROS generation, lipid peroxidation, and protein carbonylation levels [125]. Treatment of naringenin to iron overloaded mice results in a decrease in ROS generation and ROS-associated products (lipid peroxidation and protein carbonylation) [125]. This may be in part to naringenin’s primary antioxidant properties as naringenin does not raise levels of antioxidant proteins such as catalase and SOD [125].

Naringenin, a flavonoid found mostly in citrous fruits, may directly bind to Fe^2+^ ions and act as a metal chelator. Another property Naringenin possesses is that it can restore decreases in AChE activity, therefore if characteristics such as spatial memory and learning were assessed naringenin may have negative effects in this regard.

Flavonoid compounds derived from the styphnolobium japonicum have been shown to protect nematode worms from aberrant ROS generation [104]. Namely, quercetin, kaempferol, and genistein contribute to the elimination of ROS. Juglone is a pro-oxidant and is fatal in small quantities to nematode worms. The addition of flavones present in s. japonicum protects nematode worms from juglone-induced mortalities [104]. Flavones of s. japonicum protects nematode worms by inducing activation of skinhead transcription factor 1 (SKN-1) which in turn increases Nrf2 signalling [126]. Nrf2 signalling is already known to induce increased SOD expression and reduced neuroinflammatory response through reducing inflammatory cytokines such as IL-1 and TNFα [115]. Although unmeasured protective properties of s. japonicum may come from increased SOD expression and decreased inflammatory response [104]. When SKN-1 function is impaired the protective effects of flavones present in s. japonicum is completely abolished. Lastly, Flavones of s. japonicum reduce α- synuclein aggregation likely by reducing the effects of aberrant ROS generation which exacerbates α- synuclein aggregation [40,41,104].

Antioxidant treatments in animal models of aberrant ROS production, AD, and PD show that these treatments can provide protective benefits beyond cell models. The mechanisms of how antioxidants protect animals from OS are largely like those seen in cell models. Animal models have a CNS system with aspects similar to humans, for example, the dopaminergic system, therefore intact dopaminergic systems provide another level of evidence for translating these results to humans. The primary and secondary antioxidant properties of exogenous protect animals from aberrant ROS damage and significant neurodegeneration, as seen through rescued behavioural symptoms including memory and motor systems.

Antioxidant enzymes clearly play a significant role in maintaining safe levels of OS in biological systems, and it is shown that increased expression of this enzyme is protective against OS in mice. Causing direct damage to DNA, proteins and lipids Several phenotypes are identified where the loss of SOD-2 expression is associated with pathology of disease [33,107]. Further to this, memory deficits were prevented in mice where a reduction of cortex and hippocampal superoxide radicals was reduced [33,107,108]. The loss of manganese superoxide dismutase (SOD-2) expression has been observed in humans in an age-related pattern reducing the physiological defence against ROS [109].

### 3.3. Antioxidant Treatment in Human Models with Alzheimer’s and Parkinson’s Disease

Currently, the diversity of antioxidant compounds in human trials for preventing or slowing down AD and PD has been limited, of note resveratrol and vitamin E have been extensively studied for their neuroprotective roles [98,127,128,129,130]. Patients with mild to moderate dementia due to AD were treated with resveratrol to assess its neuroprotective effects. It was outlined that resveratrol was a safe therapeutic with no risk of increased adverse events such as nervous system disorders, gastrointestinal disorders, and psychiatric disorders [128]. Treatment with resveratrol was not able to reduce Aβ40 levels at a rate quicker than the placebo group in both plasma and CSF, there was no significant change in reduction of Aβ42 between resveratrol and the placebo group [128]. Patients treated with resveratrol had significantly more neurodegeneration indicated as an increase in ventricular volumes and greater change in brain volume, which may have occurred from increased Aβ40 levels [128]. On the other hand, a later study investigating the effects of resveratrol outlined those patients treated with resveratrol had significant reductions in Aβ40 levels and overall attenuation of neuroinflammation [127]. Resveratrol was sufficient to reduce TNFα levels and increase anti-inflammatory cytokines such as IL-4. After 52 weeks of resveratrol treatment patients had no significant drop in MMSE scores whilst patients on the placebo had significant drops, on the other ADLs were significantly impaired to the same extent as the placebo group [127]. The lack of beneficial therapeutic effects from resveratrol may be due to lower bioavailability in the CNS as perhaps it can’t penetrate the BBB as well as antioxidants in animal models.

Vitamin E treatment effects to slow or prevent AD have been widely studied with differing results [96,98,129,130]. Early evidence of the neuroprotective properties of vitamin E showed that event-free survival was increased with a median survival of an extra 230 days compared to the placebo [109]. As a result, scores on the blessed dementia scale and dependence scale were significantly reduced however other measures of the severity of AD including MMSE UPDRS were not significantly different [96]. Conversely, another study showed that vitamin E treatment was unable to significantly decrease the chance of patients with MCI progressing to AD. Vitamin E treatment had little effect on the cognitive domains of patients with MCI or who had progressed to AD [98]. Building upon this Lloret et al. outlined two groups of patients when treated with vitamin E: those who respond and those who are unresponsive. Vitamin E treatment in patients with AD showed exacerbated decline when treated with vitamin E whilst another group showed significant differences to the non-respondents. In this study in the respondent group, it was shown that vitamin E was able to reduce products of OS such as MDA and oxidised glutathione levels [129]. Lastly, a more recent study outlined that vitamin E treatment significantly decreased the rate of decline of scores on ADLs [129]. However, these were not recapitulated on other cognitive tests [129]. These conflicting results may be from a lack of standardised protocol as most experiments use different concentrations to each other: Sano et al.; 1000 IU, Peterson et al.; 2000 IU, Lloret et al.; 800IU and Dysken et al. 2000 IU [96,98,129,130]. In excess concentrations, some antioxidants display cytotoxic properties and in too low concentrations they cannot mount an optimal response to OS. Using a standardised concentration would eliminate these concerns.

Although no clinical trials have been done to assess curcumins effect on people afflicted with AD and PD it has been investigated for its antioxidant capabilities in patients with type II diabetes [131]. In this trial curcumin significantly raised levels of SOD in both males and females resulting in a significant reduction of OS biomarkers [131]. In another clinical trial curcumin treatment was shown to attenuate OS induced by radioactive iodine in treating thyroid cancer [132]. Curcumin was able to significantly reduce the amount of double strand breaks in human blood exposed to radioactive iodine. Considering cell and animal models curcumin may be a promising agent in reducing ROS in AD and PD. This final piece of evidence showing the enhancement of antioxidant defences in humans provides strong evidence for its efficacy. However, other measures would need to be included to cement its use in treating AD and PD including curcumins bioavailability in the brain, subsequent measures of ROS levels, additional antioxidant proteins, neurodegeneration, and Aβ and α-synuclein levels.

The administration of SOD-2 supplementation is not common in practice for the treatment of disorders where the pathogenesis of disease is associated with OS. There are complications for oral administration due to the protein being digested in the gut before it is able to act systemically as an antioxidant enzyme. A number of trials have been carried out for AD and PD with few positive results and a selection of some of these trials are in Table 2 [133,134,135,136,137,138,139]. One reason for the failure of these antioxidant therapies may be that their effect is downstream of the ROS production, particularly in the mitochondria. Targeting superoxide free radicals in the mitochondria with supplemental or enhanced expression of SOD2 may address this lack of antioxidant activity and restore the oxidative homeostasis in tissues with OS, particularly in brain tissue.

Further, understanding of the role of SOD-2 function, in reducing OS in humans needs to be better defined, especially in relation to neurodegenerative disorders like AD. New SOD-2 mimetics have been developed to reduce OS after radiotherapy in cancer patients, Avasopasem Manganese (GC4419 AVA), that selectively reduces superoxide dismutase and peroxide. In patients [110]. Could exogenous supplementation of SOD-2 be administered in humans to reduce the OS that causes neurodegeneration and slow the progression of PD and AD? [13].

## 4. Future Perspectives

New regimens to reduce oxidative damage from lifestyle may decelerate the progression of AD. Due to the dissonance between the success of antioxidant treatment in cells and animals versus the conflicting results present in human trials a standardised protocol focussing on antioxidants that have shown consistent success in cell and animal models may be the way forward. For example, curcumin, quercetin, metal chelators, and carotenoids show evidence that they can reduce protein aggregation and protect neurons against aberrant ROS levels. Therefore, assessing if these compounds retain these effects in humans would be the next step forward.

Innovative clinical trials could demonstrate how new therapies can enhance antioxidant function of mitochondrial antioxidant enzymes, like SOD-2. Some antioxidant mimetics have been developed for other treatment purposes for some patients to reduce oxidative damage during radiotherapy treatment with AD and these products could be repurposed for reducing oxidative stress in neurodegenerative disease. Proof of concept could be found by using brain organoids in experiments to demonstrate that the reduction of ROS and oxidative stress reduces neurodegeneration [13].

Research to determine detailed dietary regimens to mitigate neurodegenerative pathology for individuals, with known AD susceptibility genes, and in patients at an early stage of their diagnosis would greatly enhance the current support of these patients.

Antioxidant treatment is repeatedly demonstrated to slow the progression of AD and PD-mediated ROS generation in animal models. Although, with the inconclusive results of resveratrol and vitamin E treatment in humans several antioxidants show promising therapeutic effects in cell and animal models. Further combination therapies of antioxidant enzymes should be trialled in patient groups. Some of these regimens may be patient specific so understanding how different individuals respond to different therapies may be determined by their genotypes or phenotypes.

New trials using hormone replacement therapies could reduce the pathogenesis of AD in women, which is another discussion altogether not really addressed in this review. Due to the dissonance between the success of antioxidant treatment in cells and animals versus the conflicting results present in human trials a standardised protocol focussing on antioxidants that have shown consistent success in cell and animal models may be the way forward. For example, curcumin, quercetin, metal chelators, and carotenoids show evidence that they can reduce protein aggregation and protect neurons against aberrant ROS levels. Therefore, assessing if these compounds retain these effects in humans would be the next step forward.

Moving forward, the information in this review should be recapitulated in patients afflicted with AD and PD to cement the protective properties of these antioxidants, especially new ventures that enhance SOD-2 function, like the new mimetics of this enzyme may show promise for future defences against OS induced neurodegeneration.

## 5. Conclusions

Antioxidants provide an accessible and cost-effective way to treat AD and PD due to their abundance in herbal compounds. In cell and animal model antioxidants show strong protective properties against ROS, Aβ, and α-synuclein. Namely, quercetin, curcumin, and metal chelators which consistently target the underlying mechanism of AD and PD with success. Specifically, protective mechanisms include enhanced endogenous defences: SOD, glutathione, and catalase as well as directly neutralising ROS. As a result, downstream ROS-induced cytotoxic events such as Aβ, α- synuclein, lipid peroxidation, and DNA oxidation are attenuated. Neuron death is reduced, as shown through the ameliorated depletion of dopaminergic neurons in the SNpc. As a result of decreased neuron death in the CNS results in the rescue of spatial memory and motor function.

Not all family members with the APOE AD susceptibility genotype experience neurodegenerative pathology and mitigation of the disease have been observed with lifestyle choices, some of which enhance antioxidant function. This provides evidence that exogenous factors could improve patient outcomes with AD [141].

## Figures and Tables

**Table 1:** Summary of the protective mechanisms of certain antioxidants and the evidence for them. Collated from cell, animal and human models of AD, PD and aberrant ROS generation Abbreviations: IFNγ: Interferon-gamma, IL: Interleukin, TNFα: Tumour necrosis factor alpha, SOD: superoxide dismutase, GSH: Glutathione, Aβ: Amyloid-beta, ROS: Reactive oxygen species, Bcl-2: B-cell lymphoma 2 protein, Bax: Bcl-2-associated protein X, TH: Tyrosine Hydroxylase, SKN-1: Skinhead transcription factor 1, SIRT1: Silent information regulator 1, FoxO3a: Forkhead box class O3a, PGC1α: Peroxisome proliferator-activated receptor gamma coactivator 1 alpha, Nrf2: Nuclear Factor Erythroid 2- related Factor 2, Nrf1: Nuclear respiratory factor 1, GSSG: Glutathione disulfide, DA: Dopamine.

Antioxidant Treatment	Protective Mechanisms	Evidence of Protective Mechanisms
Quercetin	↓IFNγ, ↓IL6, ↓IL-1β, ↓TNFα, ↑SOD, ↑GSH	↓DNA oxidation, ↓Lipid peroxidation, ↓protein carbonylation, ↓α-synuclein
Curcumin	↑GSH, ↑LC3-II, ↑SIRT1, ↑SOD	↑cell viability, ↓αsynuclein, ↓lipid peroxidation, ↓DNA oxidation
Metal chelators	↑SOD, ↑catalase, ↓iron in the CNS and periphery	↑cell viability, ↓ Caspase activation, ↑TH, ↑Bcl-2
VA, TFA+ PCA	↑SOD, ↑Fox03a, ↑catalase,↑SIRT1, ↑PGC1α, ↑Nrf2	↑cell viability, ↓ROS, ↑Bcl-2
Sinapic acid	↑PGC1α, ↑Nrf2, ↑SOD, ↑catalase	↑cell viability, ↓ Caspase activation, ↓ROS, ↓DNA oxidation
17β-Estradiol	↑SIRT1, ↑PGC1α, ↑Nrf2, ↑Fox03a, ↓IL-1β, ↓TNFα,	↓DNA oxidation, ↓ROS, ↓Glia activation, ↓Aβ
Vitamin A	↑α-synuclein, ↑Aβ, ↑pTau	↑cell viability
Vitamin C	↓IL6, ↑IL-10, ↓TNFα	↑TH, ↓Glia activation
Vitamin E	ROS neutralization	↓Lipid peroxidation
Vitamin B12	↑PTBP1	↑cell viability
Carotenoids	↑GSH	↓ Caspase activation, ↓Bax, ↑Bcl-2, ↑DA, ↓Aβ
Flavonoids	ROS neutralisation, ↑SKN-1 activation, ↑Nrf2	↓αsynuclein
EGCG	↑SKN-1 activation	↓Aβ, ↓αsynuclein
Resveratrol	↓TNFα, ↓IL4	No significant reduction of ROS
SOD-2 (GC4419 AVA)	↑SOD	↑cell viability, ↓ROS, ↓lipid peroxidation, ↓DNA oxidation

**Table 2:** Antioxidant clinical trials. A selection of clinical trials of antioxidant therapies for Alzheimer’s Disease (AD) and Parkinson’s Disease (PD) patients [139].

Disorder	Results	Intervention	Author	Date
AD	No effect on AD	Curcumin	Sechi G et al. [135]	1996
AD	No beneficial effect	Vitamin E	Sies H [133]	2017
AD	No beneficial effect	Ginkgo biloba	Zoller B et al. Zimmermann et al. [136,137]	2001 2002
AD	Regulates neuroinflammation	Resveratrol	Turner R et al. [127,128]	2015 2017
AD	Improved memory and attention in nondemented adults	Curcumin	Smith et al. [138]	2018
AD	Improves cognitive impairment in mice	Beta Carotene	Hira S et al. [124]	2019
PD	Alleviated symptoms No impact on QOL	Curcumin	Sechi G et al. [135]	1996
PD	No effect on PD	Datotop isoprene tocopherol	Benfeitas R [140]	2017
PD	No reduction in risk of PD	Vitamin C, E and carotenoids	Schaar CE et al. [134]	2015
PD	No reduction in risk of PD	Vitamin C and carotenoids	Schieber M and Chanel NS [12]	2014
